# Short-term (1–3 months) versus standard (12 months) dual antiplatelet therapy following new-generation drug-eluting stent implantation: A meta-analysis of randomized controlled trials

**DOI:** 10.1097/MD.0000000000038071

**Published:** 2024-05-31

**Authors:** Penghui Xiong, Chunhua Zheng, Jianfeng Fan, Hongyu Zhang, Can Li

**Affiliations:** a Department of Cardiology, The First Hospital of Nanchang, Nanchang, Jiangxi, China.

**Keywords:** acute coronary syndrome, drug-eluting stent, dual antiplatelet therapy, percutaneous coronary intervention

## Abstract

**Background:**

Patients undergoing percutaneous coronary intervention mainly receive antiplatelet therapy. However, limited data are available regarding the optimal dual antiplatelet therapy (DAPT) following the implantation of new-generation drug-eluting stent (DES).

**Objective:**

This study aimed to compare the clinical outcomes of short-term (1–3 months) DAPT and standard (12 months) DAPT after the implantation of a new-generation of DES.

**Methods:**

We systematically searched PubMed, The Cochrane Library Database, Embase for trials that compared short-term (1–3 months) and standard DAPT after the implantation of next-generation DES were retrieved from all published studies in English until December 31, 2021. The primary endpoint was major bleeding. The secondary endpoints included all-cause mortality, cardiac death, myocardial infarction, stroke, stent thrombosis, and all bleeding.

**Results:**

This study included a total of 7 randomized controlled trials, comprising 28,344 subjects. Regarding primary endpoints, short-term DAPT exhibited a significantly lower incidence of major bleeding compared with standard DAPT [relative risk (RR): 0.66, 95% confidence interval (CI): (0.54, 0.81), *P* < .0001]. For secondary endpoints, there were significant differences between short-term and standard DAPT in all bleeding [RR: 0.59, 95% CI: (0.50, 0.69), *P* < .00001]. However, no significant differences were identified in all-cause mortality [RR: 0.96, 95% CI: (0.77, 1.18), *P* = .27], myocardial infarction [RR: 0.98, 95% CI: (0.82, 1.18), *P* = .86], cardiac death [RR: 0.83, 95% CI: (0.63, 1.10), *P* = .20], stroke [RR: 1.08, 95% CI: (0.79, 1.47), *P* = .63], cerebrovascular [RR: 1.08, 95% CI: (0.79, 1.47), *P* = .63], and stent thrombosis [RR: 1.13, 95% CI: (0.80, 1.57), *P* = .49] between the 2 groups.

**Conclusion:**

In patients undergoing implantation of a new-generation of DES, short-term (1–3 months) DAPT exhibited no inferiority compared with standard (12 months) DAPT in terms of all-cause mortality, cardiac death, myocardial infarction, stroke, and definite or probable stent thrombosis compared with standard (12 months) DAPT. However, short-term DAPT appeared superior to standard DAPT in terms of major bleeding and all bleeding.

## 1. Introduction

After coronary stent implantation, dual antiplatelet therapy (DAPT) with aspirin and a P2Y12 receptor inhibitor is well established to decrease recurrent ischemic events.^[1]^ However, the optimal duration of DAPT following percutaneous coronary intervention (PCI) remains a matter of ongoing debate.

Compared with bare-metal stent (BMS), the new-generation drug-eluting stent (DES) is associated with lower incidence rates of stent restenosis, myocardial infarction, and revascularization. Moreover, studies indicated that extending DAPT time does not reduce the risk of thrombotic events, while it increases the risk of bleeding.^[2,3]^ Therefore, it is essential to explore how to shorten the duration of DAPT. To date, numerous medium-sized randomized trials have compared DAPT with different durations and used multiple DESs, and the outcomes did not indicate the advantages of either standard or extended treatment.^[4,5]^

In addition, numerous disadvantages of standard or long-term DAPT have been found, mainly including high bleeding risk and high treatment cost, thus, it is necessary to weigh the disadvantages and potential benefits of standard or long-term DAPT. A great number of clinical experiments have demonstrated that the new-generation DES is associated with a lower risk of ST formation compared with the first-generation DES or BMS.^[6,7]^ Moreover, with the improvement of interventional techniques, interventional devices, and other related factors, it seems reasonable to shorten the duration of DAPT. In recent years, numerous extensive clinical studies have examined the differences between short-term (1–3 months) and standard (12 months) DAPT in patients undergoing new-generation DES implantation.^[8,9]^ However, clear conclusions remain elusive. Hence, the objective of this study was to conduct a comparative analysis between short-term (1–3 months) and standard (12 months) DAPT following new-generation DES implantation and to conduct a meta-analysis to provide reference for the formulation of clinical DAPT treatment plan in the future.

## 2. Methods

### 2.1. Data sources and search strategy

We searched PubMed, Embase, and the Cochrane Library databases were searched for retrieving studies that compared short-term (1–3 months) and standard (12 months) DAPT. It was also attempted to search for relevant trials in the ClinicalTrials.gov. Search terms included “dual antiplatelet therapy,” “DAPT,” “drug e-eluting stents,” “DES,” “percutaneous coronary intervention,” and “acute corona syndrome.” The search period involved the inception of the databases until December 31, 2021. Only studies published in the English language were considered for inclusion in the analysis. Ethical approval and patient consent were not required because this is an analysis of previously published studies.

### 2.2. Study selection

#### 2.2.1. Inclusion criteria

The inclusion criteria were summarized as follows: 1. Randomized controlled trials (RCTs); 2. Studies that compared short-term (1–3 months) and standard (12 months) DAPT; 3. Trials on implanted with new-generation DES; 4. Trials reporting clinical outcomes in patients, including major bleeding, all bleeding events, all-cause mortality, myocardial infarction, cardiac death, stroke, and definite or probable stent thrombosis.

#### 2.2.2. Exclusion criteria

The exclusion criteria were summarized as follows 1. Reviews, case reports, letters, ongoing trials or editorials; 2. Duplicate publications; 3. Studies that enrolled patients undergoing first-generation DES or first-generation BMS implantation; 4. Studies that could meet the inclusion criteria, while no experimental data could be extracted.

### 2.3. Study endpoints

The main endpoint of this study was major bleeding. Secondary endpoints included all bleeding, all-cause mortality, myocardial infarction, cardiac death, stroke, and definite or possible stent thrombosis. The definitions for all clinical endpoints adhered to the original criteria outlined in the included studies. Due to variations in bleeding definitions across experiments, inclusion data adhered to the thrombolysis in myocardial infarction bleeding grading standard, Bleeding Academic Research Consortium bleeding grading standard, and GUSTO bleeding grading standard.^[10]^

### 2.4. Data extraction and risk of bias assessment

Two researchers (XPH and ZCH) independently completed the study selection and quality evaluation of the retrieved articles, removing those that did not meet the eligibility criteria based on their titles and abstracts. Thereafter, according to the eligibility criteria, the full text of the article was studied and evaluated. Where there were discrepancies in 2 researchers’ assessment, a third researcher (LC) was involved, and consensus was reached. Subsequently, relevant data were extracted from the studies, including study characteristics, patients’ baseline characteristics, and clinical outcomes. The Cochrane Collaboration’s Risk of Bias Tool was utilized to assess the quality of the included studies.

### 2.5. Statistical analysis

Statistical analysis was performed using RevMan 5.3 software. Binary variables were expressed by relative risk (RR) and 95% confidence interval (CI). Heterogeneity was quantitatively evaluated for the included studies using the *I*^2^ statistic. If the *I*^2^ < 50%, indicating small or no heterogeneity, a fixed-effects model was employed for statistical analysis. If the heterogeneity test *I*^2^ ≥ 50%, a random-effects model was used for data analysis.

## 3. Results

### 3.1. Search results and study characteristics

**Figure 1.**
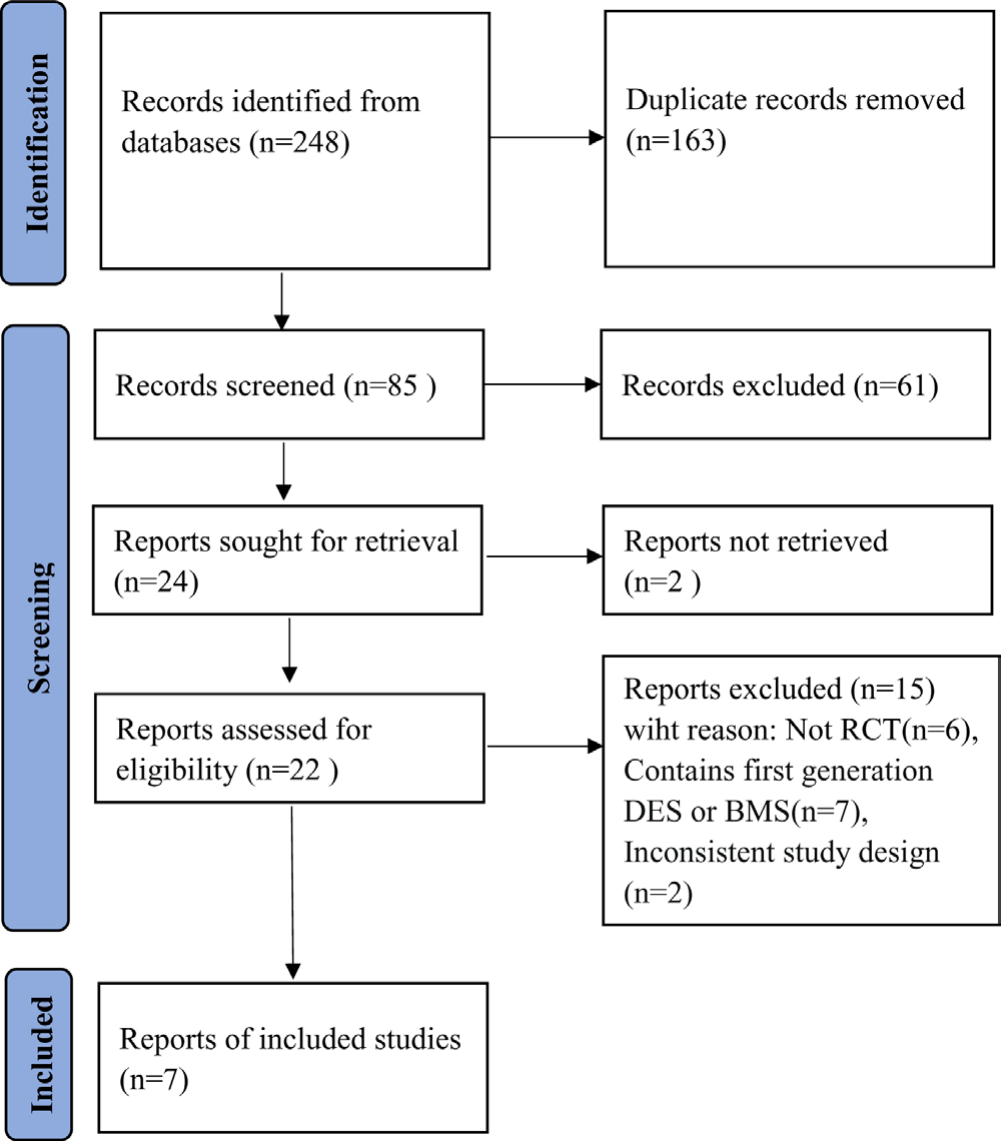
Flowchart for study selection.

According to the search strategy, a total of 248 documents were obtained. After eliminating the duplicate publications, there were 85 articles, and 24 articles remained after reading the titles and abstracts. The full-text of remaining articles was read and 7 articles were finally involved according to the selection criteria.^[9,11–16]^ The study selection process is illustrated in Figure 1.

In order to reduce the heterogeneity among studies and the potential risk of bias, the present meta-analysis included data from the 1-year endpoints of the GLASSY and REDUCE trials. The study involved 7 trials, involving a total of 28,344 subjects. Upon examining participants’ baseline characteristics and clinical manifestations, 14,163 patients were assigned to the short-term group, while 14,181 patients were allocated to the standard group.

### 3.2. Patients’ characteristics of included studies

Baseline characteristics and clinical manifestations included trial name, duration of DAPT, year of publication, study population, stent type, average age, male: female ratio, follow-up time, average body mass index, smoking status, diabetes, hypertension, dyslipidemia, average left ventricular ejection fraction, etc (Table 1).

**Table 1:** Characteristics of the included trials.

Name of experiment	OPTIMIZE		REDUCE		GLASSY		SMART-CHOICE		STOPDAPT-2		TWILIGHT		TICO	
Duration of dual antiplatelet therapy	3	12	3	12	1	12	3	12	3	12	3	12	1	12
Total number	1563	1556	751	745	3794	3791	1495	1498	1500	1509	3555	3564	1527	1529
Time of publication	2013		2019		2019		2019		2019		2019		2020	
Type of study	RCT		RCT		RCT		RCT		RCT		RCT		RCT	
countries	Brazil		Multiple countries		Multiple countries		Korea		Japan		Multiple countries		Korea	
Study population	ACS+ SCAD		ACS		ACS+ SCAD		ACS+ SCAD		ACS+ SCAD		ACS+ SCAD		ACS	
Type of Stent	ZES		new generation DES		BES		EES/BES		EES		new generation DES		BES	
Mean age	61.3 (10.4)	61.9 (10.6)	61 (8)	60 (8)	64.9 (10.3)	64.8 (10.3)	64.6 (10.7)	64.4 (10.4)	68.1 (10.9)	69.1 (10.4)	65.2 (10.3)	65.1 (10.4)	61 (11)	61 (11)
Proportion of males	992 (63.5)		620 (82.6)		2884 (76.0)		1087 (72.7)		1183 (78.9)		2709 (76.2)		1204 (78.8)	
Duration OF FOLLOW-UP	12		24		24		12		12		12		12	
Mean BMI	–	–	–	–	28.0 (4.5)	27.9 (4.5)	24.5 (3.1)	24.7 (3.2)	24.4 (3.5)	24.4 (3.5)	28.6 (5.5)	28.5 (5.6)	24.9 (3.2)	24.9 (3.3)
Current Smoking	290 (18.6)	269 (17.3)	313 (42.1)	314 (42.7)	1084 (28.6)	1102 (29.1)	424 (28.4)	367 (24.5)	399 (26.6)	311 (20.6)	726 (20.4)	822 (23.1)	555 (36.3)	587 (38.3)
Diabetes	554 (35.4)	549 (35.3)	162 (21.6)	145 (19.5)	923 (24.3)	899 (23.7)	570 (38.2)	552 (36.8)	585 (39.0)	574 (38.0)	1319 (37.1)	1301 (36.5)	418 (27.4)	417 (27.3)
Hypertension	1350 (86.4)	1371 (88.2)	379 (50.7)	375 (50.7)	2752 (72.5)	2740 (72.3)	921 (61.6)	919 (61.3)	1105 (73.7)	1116 (74.0)	2580 (72.6)	2574 (72.2)	760 (49.8)	781 (51.1)
Dyslipidemia	953 (63.2)	953 (63.2)	346 (46.3)	333 (44.9)	2402 (63.3)	2476 (65.3)	673 (45.1)	679 (45.5)	1116 (74.4)	1128 (74.8)	2157 (60.7)	2146 (60.2)	924 (60.5)	922 (60.3)
Mean LVEF	–	–	–	–	55.1 (11.8)	55.3 (11.3)	60.0 (10.9)	59.9 (10.7)	59.8 (10.2)	59.7 (10.6)	–	–	–	–
Previous MI	541 (34.6)	542 (34.8)	–	–	869 (22.9)	893 (23.6)	62 (4.1)	65 (4.3)	207 (13.8)	199 (13.2)	1020 (28.7)	1020 (28.6)	64 (4.2)	49 (3.2)
Previous CABG	111 (7.1)	128 (8.2)	21 (2.8)	21 (2.8)	204 (5.4)	239 (6.3)	–	–	17 (1.1)	42 (2.8)	362 (10.2)	348 (9.8)	8 (0.5)	10 (0.7)
Previous PCI	327 (20.9)	297 (19.1)	88 (11.7)	73 (9.8)	1236 (32.6)	1286 (33.6)	–	–	503 (33.5)	529 (35.1)	1502 (42.3)	1496 (42.0)	135 (9)	127 (8)
SCAD	935 (59.8)	911 (58.6)	–	–	1855 (48.9)	1890 (49.9)	625 (41.8)	625 (41.8)	935 (62.3)	926 (61.4)	1281 (36.1)	1222 (34.7)	–	–
UA	–	–	114 (15.2)	103 (13.8)	490 (12.9)	499 (13.2)	467 (31.2)	491 (32.8)	193 (12.9)	214 (14.2)	1249 (35.1)	1245 (34.9)	442 (29)	484 (32)
NSTEMI	84 (5.4)	84 (5.4)	267 (35.6)	305 (41.0)	760 (20)	737 (19.4)	239 (16)	230 (15.4)	81 (5.4)	99 (6.6)	1024 (28.8)	1096 (30.8)	539 (35)	488 (32)
STEMI	–	–	370 (49.3)	336 (45.2)	689 (18.2)	665 (17.5)	164 (11)	150 (10)	291 (19.4)	270 (17.9)	–	–	546 (36)	557 (36)

ACS = acute coronary syndrome, BES = biomolimus-eluting stent, BMI = body mass index, CABG = coronary artery bypass, DES = drug-eluting stent, EES = eviolimus-eluting stent, LVEF = left ventricular ejection fraction, MI = myocardial infarction, NSTEMI = non ST segment elevation myocardial infarction, PCI = percutaneous coronary intervention, RCT = randomized controlled trial, SCAD = stable coronary artery disease, STEMI = ST segment elevation myocardial infarction, UA = unstable angina pectoris, ZES = zotamolimus-eluting stent.

### 3.3. Risk of bias assessment and sensitivity analysis

**Figure 2.**
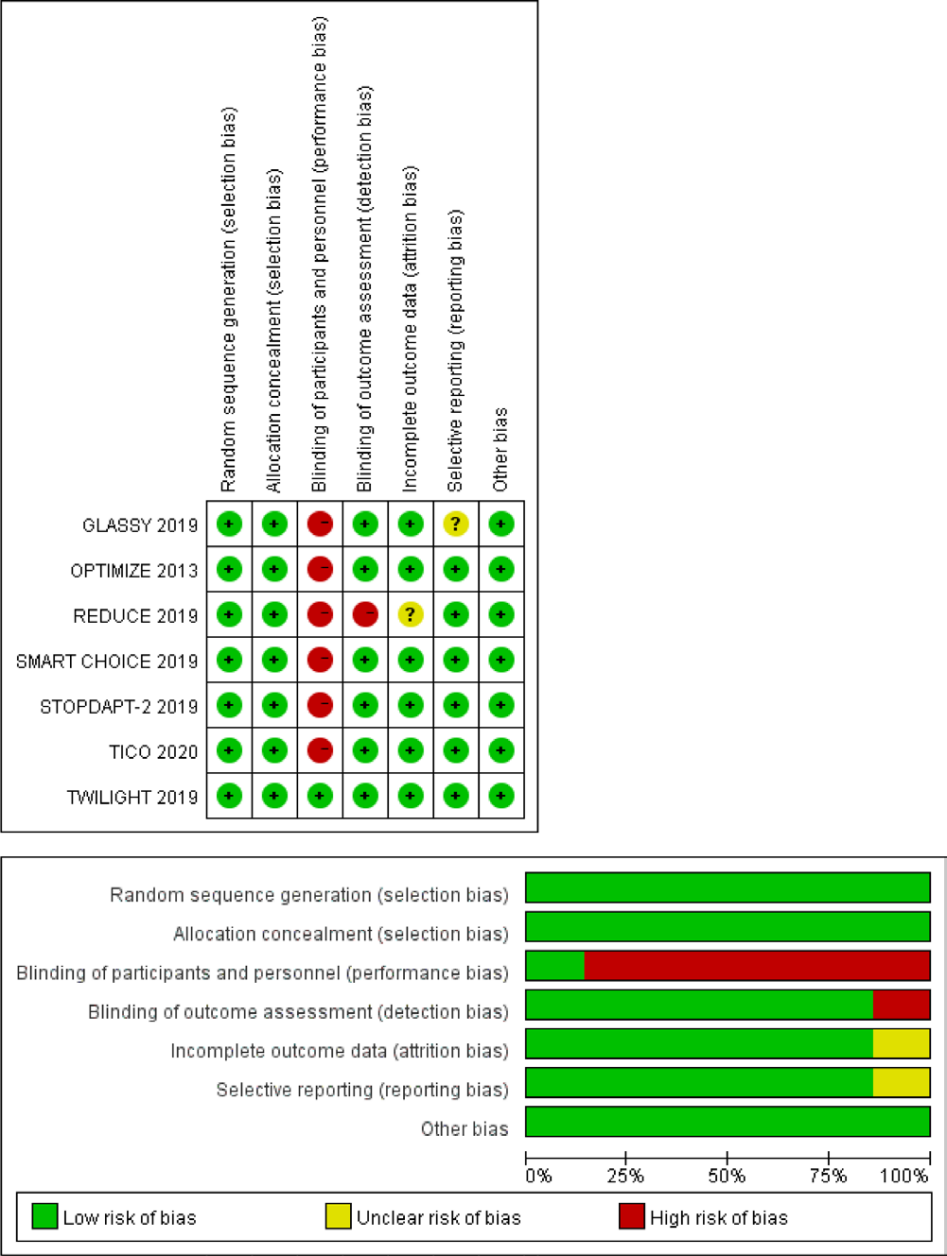
Risk of bias graph and summary.

The Cochrane Collaboration’s Risk of Bias Tool was used to evaluate the quality of the included studies, and the results of quality evaluation are displayed in Figure 2. Although there were 6 non-double-blinded, RCTs and only 1 double-blinded RCT, all clinical outcomes were clearly defined and determined by the blind method, and the open-label trial design should not be considered a notable source of bias. Because <10 articles were involved in this study, no risk of publication bias was evaluated. Sensitivity analysis tests the robustness of the results by excluding 1 study at a time. Sensitivity analysis indicated that the heterogeneity of major bleeding events was significantly reduced when the GLASSY study was excluded, and the results were still statistically significant.

### 3.4. Clinical endpoints

#### 3.4.1. Major bleeding

**Figure 3.**
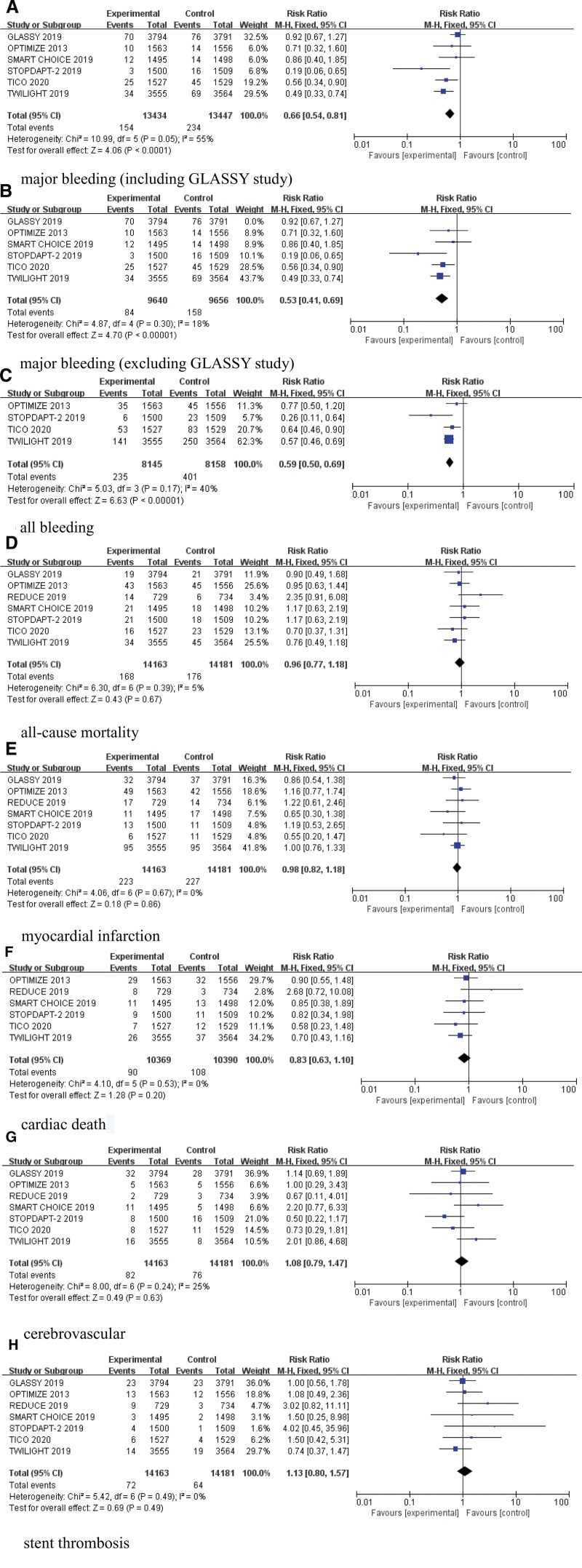
Forest plot of major bleeding, all bleeding, all-cause mortality, myocardial infarction, cardiac death, cerebrovascular, stent thrombosis.

In this study, 6 trials reported major bleeding events in a total of 26,881 patients. Heterogeneity test revealed that there was a significant heterogeneity between the 2 groups (*I*^2^ = 55%). When reanalyzing the studies, it was found that the GLASSY study exhibited selectivity in the control group, with patients using P2Y12 inhibitors. Those with stable coronary artery disease (SCAD) were prescribed clopidogrel, while those with acute coronary syndrome (ACS) were administered ticagrelor. This differential treatment approach may contribute to a reduction in the risk of major bleeding. Consequently, the primary source of heterogeneity could be attributed to the GLASSY study. When the GLASSY study was removed and sensitivity analysis was conducted, *I*^2^ decreased from 55% to 18%, and *P*-value increased to 0.3. The results indicated that the difference was statistically significant [RR: 0.53, 95% CI: (0.41, 0.69), *P* < .00001] (Fig. 3A and B).

#### 3.4.2. All bleeding

There was a significant difference in all bleeding between the short-term DAPT group and the standard DAPT group [RR: 0.59, 95% CI: (0.50, 0.69), *P* < .00001, *I*^2^ = 40%] (Fig. 3C).

#### 3.4.3. All-cause mortality

All trials reported the incidence of all-cause mortality. There was no significant difference in all-cause mortality between the short-term DAPT group and the standard DAPT group [RR: 0.96, 95% CI: (0.77,1.18), *P* = .67, *I*^2^ = 5%] (Fig. 3D).

#### 3.4.4. Myocardial infarction

No significant difference was found in myocardial infarction between the short-term DAPT group and the standard DAPT group [RR: 0.98, 95% CI: (0.82, 1.18), *P* = .86, *I*^2^ = 0%] (Fig. 3E).

#### 3.4.5. Cardiac death

Only 6 trials reported the incidence of cardiac death. There was no statistically significant difference in the risk of cardiac death between the short-term DAPT group and the standard DAPT group [RR: 0.83, 95% CI: (0.63, 1.10), *P* = .20, *I*^2^ = 0%] (Fig. 3F).

#### 3.4.6. Cerebrovascular

There was no statistically significant difference in cerebrovascular events between the short-term DAPT group and the standard DAPT group [RR: 1.08, 95% CI: (0.79, 1.47), *P* = .63, *I*^2^ = 25%] (Fig. 3G).

#### 3.4.7. Stent thrombosis

All trials reported the incidence of stent thrombosis, and no statistically significant difference was found between the short-term DAPT group and the standard DAPT group [RR: 1.13, 95% CI: (0.80, 1.57), *P* = .49, *I*^2^ = 0%] (Fig. 3H).

## 4. Discussion

This study included 7 RCTs with a total of 28,344 patients. The results indicated that short-term (1–3 months) DAPT was not inferior to standard (12 months) DAPT in terms of all-cause mortality, cardiac death, myocardial infarction, stroke, and stent thrombosis. Besides, the former is superior to the latter in terms of risk of major bleeding and all bleeding events.

The guidelines of European Society of Cardiology/European Society of Cardiothoracic Surgery (ESC/EACTS) and American college of cardiology/American Heart Association (ACC/AHA) recommend that DAPT should be taken for at least 6 months for SCAD patients undergoing DES and for at least 12 months for ACS patients receiving DES implantation.^[17,18]^ However, as an important risk factor for stent thrombosis, the stent type is closely associated with the duration of DAPT. Compared with the first-generation DES, the new-generation DES mainly includes everolimus-eluting stent, zotarolimus-eluting stent, and biodegradable polymer drug-eluting stent. The everolimus-eluting stent and zotarolimus-eluting stent mainly use cobalt-chromium alloy as the scaffold material, because it has higher density and strength, making the scaffold pillar thinner. The second representative surface carries biocompatible polymers, involving choline phosphate and fluoropolymer. Although they are undergradable, they alleviate the inflammatory reaction compared with the first-generation DES.^[19,20]^ Furthermore, the second-generation DES exhibits enhanced lipophilicity, resulting in pronounced local effects of the drug. On the other hand, the third-generation degradable polymer DES serves an antiproliferative function postimplantation. The polymer on its surface undergoes complete degradation within 6 to 9 months following implantation, leaving bare metal stents. This degradation minimizes allergic reactions caused by polymer carriers. A growing body of clinical evidence revealed that the risk of stent thrombosis in the early, late, and very late stages of the new-generation DES was lower, and the risk of stent thrombosis was about half that of the first-generation DES. Moreover, with the development of interventional devices and the improvement of clinicians’ interventional skills, several clinical trials have studied the safety and efficacy of short-term DAPT. The results of these studies appeared optimistic, while shortening the duration of DAPT remains essential.

TWILIGHT study mainly included high-risk SCAD and ACS patients, and compared the efficacy of 3-month DAPT with 12-month DAPT. The results indicated that 3-month DAPT was not inferior to the 12-month DAPT in terms of all-cause mortality, myocardial infarction, cardiogenic death, stroke, stent thrombosis, and all bleeding.^[9]^ TICO study included 3056 patients with ACS, and compared the efficacy and safety of 3-month DAPT with those of 12-month DAPT following implantation of new-generation DES. The results demonstrated that 3-month DAPT was superior to the 12-month DAPT in terms of major bleeding and all bleeding events, while it was not inferior to the 12-month DAPT in terms of all-cause mortality, myocardial infarction, cardiogenic death, stroke, and stent thrombosis.^[11]^ STOPDAPT-2 study compared the efficacy of 1-month DAPT with that of 12-month DAPT in patients with SCAD and ACS. The results showed that 1-month DAPT was superior to 12-month DAPT in terms of all major bleeding events, whereas not inferior to 12-month DAPT in all-cause mortality, myocardial infarction, cardiogenic death, stroke, and stent thrombosis.^[16]^ SMART-CHOICE study comparing 1-month DAPT with 12-month DAPT involved 3119 patients, and the results showed that DAPT was not inferior to standard DAPT in terms of all-cause mortality, myocardial infarction, cardiogenic death, stroke, stent thrombosis, and massive bleeding 3 months after implantation of the new-generation DES.^[12]^ GLASSY study, OPTIMIZE study, and REDUCE study all concluded that the DAPT for patients undergoing the new-generation DES is comparable to, or not inferior to, the DAPT administered for 12 months post-implantation.^[13–15]^ The results of the present study are basically consistent with those of the abovementioned studies, however, additional studies are required to indicate whether short-term DAPT can improve patients’ prognosis.

The optimal duration of DAPT depends primarily on the dynamic balance between bleeding risk and ischemic benefit.^[21]^ In our study, the results revealed that compared with the standard DAPT, the short-term DAPT could reduce the risk of major bleeding and all bleeding events, which is consistent with other research results.^[10,22–24]^ However, there was a significant heterogeneity in the results of major bleeding risks. Sensitivity analysis indicated that the heterogeneity was significantly reduced after the exclusion of the GLASSY study, and the results demonstrated that the main bleeding risk was still statistically significant. For the heterogeneity of the GLASSY study, the greater heterogeneity could be attributed to patients’ clinical stratifications in the control group, arising from the lower bleeding risk in SCAD patients, and the utilization of stronger antiplatelet inhibitors may increase the bleeding risk.

The traditional view is that patients with ACS need long-term DAPT. However, in both the TICO and REDUCE trials, concentrating on patients with ACS who underwent new-generation DES implantation, the findings indicated that short-term DAPT resulted in a decrease in the incidence of bleeding events without a concurrent rise in the incidence of ischemic events. Therefore, it seems feasible to shorten the duration of DAPT even for ACS patients. In addition, the duration of DAPT may vary in patients with hypertension, chronic kidney disease, or diabetes. Therefore, it is crucial to consider the net benefit of the risk of ischemic and bleeding events in patients with coronary heart disease. Prioritizing the reduction of the risk of bleeding by shortening the duration of DAPT may be more significant than further reduction of the risk of stent thrombosis through prolonged DAPT duration. Further research is essential to confirm the optimal duration of DAPT in these patients. In order to reduce the occurrence of cardiovascular events and improve patients’ prognosis following PCI, a standard scoring system is required to formulate an optimal DAPT scheme for patients following PCI. At present, the PRECISE-DAPT is the sole scoring system that simplifies the assessment of short-term or standard/extended DAPT. Comprising 5 parameters (age, creatinine clearance rate, hemoglobin, white blood cell count, and previous spontaneous bleeding history), it evaluates the risk of out-of-hospital bleeding in patients undergoing DAPT.^[25]^ A score of ≥ 25 indicates a high risk of bleeding risk, recommending a 3–6 month duration for short-term DAPT. For those with a score < 25, standard/extended DAPT duration is suggested. While the PLATO test and Bern PCI test have been used for validation, prospective validation in RCTs is lacking for the PRECISE-DAPT score. Further validation is required to determine its ability to improve patients’ prognosis.^[26]^ Moreover, the data of precision-DAPT scoring research mainly originated from European and American countries. Because of the differences between European and American ethnic groups and Asian ethnic groups, precision-DAPT scoring is not necessarily appropriate for Asians. According to statistics, under the condition of receiving the same antiplatelet drugs, the risk of bleeding in Asians was significantly higher than that in Europeans and Americans.^[27]^ Among 7 studies involved in this meta-analysis, namely SMART-CHOICE, STOPDAPT-2, and TICO, all involved Asian population. The study outcomes demonstrated that short-term DAPT could be superior to or non-inferior to standard DAPT. Therefore, there is a need to develop a scoring system for the Asian population to effectively balance the risks of ischemia and bleeding. This may facilitate the implementation of optimal individualized treatments.

## 5. Limitations

This study has some limitations. Firstly, in terms of methods, the duration of DAPT was different. The duration of DAPT in the OPTIMIZE, REDUCE, SMART-CHOICE, and TICO studies was 3 months, while it was only 1 month in the STOPDAPT-2 and GLASSY studies. While only 1-year data from the GLASSY and REDUCE were included, the 2-year data were omitted, and this decision was made to maintain consistency in the timeframe of the analysis. Secondly, the use of P2Y2 receptor antagonists was different among different research groups. The new P2Y2 inhibitor is a stronger and faster antiplatelet aggregation drug. However, some studies have confirmed that compared with clopidogrel, the new P2Y2 inhibitor significantly reduced the incidence of stent thrombosis, all-cause mortality, and myocardial infarction, while the bleeding risk increased.^[28]^ There are some differences in the use of P2Y12 inhibitors in DAPT in the early stage, which could also be an important limitation of this study. Thirdly, although all the included studies utilized a new generation of DES, including the second-generation DES and the third-generation biodegradable biopolymer Morse-eluting stent and combined stent, there were differences among the stents, which could be an important limitation of this study. Fourthly, bleeding was defined differently among different studies, and the conclusion obtained by combining all the research results in this study might be biased. Because there is no uniform definition for high-risk or complex PCI, the results of this study might not be extended to complex and high-risk patients.

## 6. Conclusions

Compared with the standard (12 months) DAPT, the short-term (1–3 months) DAPT was not inferior in terms of all-cause mortality, cardiac death, myocardial infarction, stroke, and definite or stent thrombosis, while it was superior to standard DAPT in major bleeding and all bleeding events. However, due to the limitations of this study, additional RCTs are required to verify the results.

## Acknowledgments

We would like to thank all the authors in particular.

## Author contributions

**Conceptualization:** Jianfeng Fan.

**Data curation:** Penghui Xiong, Chunhua Zheng, Hongyu Zhang.

**Formal analysis:** Penghui Xiong, Chunhua Zheng, Hongyu Zhang.

**Investigation:** Penghui Xiong, Chunhua Zheng, Can Li.

**Methodology:** Penghui Xiong.

**Supervision:** Penghui Xiong, Chunhua Zheng, Can Li.

**Visualization:** Jianfeng Fan, Penghui Xiong.

**Writing – original draft:** Penghui Xiong, Chunhua Zheng.

**Writing – review & editing:** Penghui Xiong, Chunhua Zheng.
